# Identification of Blood-Based Biomarkers for the Prediction of the Response to Neoadjuvant Chemoradiation in Rectal Cancer

**DOI:** 10.3390/cancers13143642

**Published:** 2021-07-20

**Authors:** Delphine Dayde, Jillian Gunther, Yutaka Hirayama, David C. Weksberg, Adam Boutin, Gargy Parhy, Clemente Aguilar-Bonavides, Hong Wang, Hiroyuki Katayama, Yuichi Abe, Kim-Anh Do, Kazuo Hara, Takashi Kinoshita, Koji Komori, Yasuhiro Shimizu, Masahiro Tajika, Yasumasa Niwa, Y. Alan Wang, Ronald DePinho, Samir Hanash, Sunil Krishnan, Ayumu Taguchi

**Affiliations:** 1 Department of Translational Molecular Pathology, The University of Texas MD Anderson Cancer Center, Houston, TX 77030, USA; delphine.dayde@inserm.fr (D.D.); gparhy@deloitte.com (G.P.); 2 Department of Radiation Oncology, The University of Texas MD Anderson Cancer Center, Houston, TX 77030, USA; JGunther@mdanderson.org (J.G.); weksbergdc@upmc.edu (D.C.W.); Krishnan.Sunil@mayo.edu (S.K.); 3 Department of Endoscopy, Aichi Cancer Center Hospital, Nagoya 464-8681, Japan; yhirayama@aichi-cc.jp (Y.H.); mtajika@aichi-cc.jp (M.T.); yniwa@aichi-cc.jp (Y.N.); 4 UPMC Pinnacle Radiation Oncology, Harrisburg, PA 17109, USA; 5 Department of Cancer Biology, The University of Texas MD Anderson Cancer Center, Houston, TX 77030, USA; adam@glympsebio.com (A.B.); yalanwang@mdanderson.org (A.Y.W.); rdepinho@mdanderson.org (R.D.); 6 Department of Biostatistics, The University of Texas MD Anderson Cancer Center, Houston, TX 77030, USA; jaguil21@its.jnj.com (C.A.-B.); kimdo@mdanderson.org (K.-A.D.); 7 Department of Clinical Cancer Prevention, The University of Texas MD Anderson Cancer Center, Houston, TX 77030, USA; wh@cwmda.com (H.W.); HKatayama1@mdanderson.org (H.K.); SHanash@mdanderson.org (S.H.); 8 Hangzhou Cosmos Wisdom Mass Spectrometry Center of Zhejiang University Medical School, Hangzhou 311200, China; 9 Division of Molecular Diagnostics, Aichi Cancer Center, Nagoya 464-8681, Japan; y.abe@aichi-cc.jp; 10 Department of Gastroenterology, Aichi Cancer Center Hospital, Nagoya 464-8681, Japan; khara@aichi-cc.jp; 11 Department of Gastroenterological Surgery, Aichi Cancer Center Hospital, Nagoya 464-8681, Japan; t-kinoshita@aichi-cc.jp (T.K.); kkomori@aichi-cc.jp (K.K.); yshimizu@aichi-cc.jp (Y.S.); 12 Department of Radiation Oncology, Mayo Clinic Florida, Jacksonville, FL 32224, USA; 13 Division of Advanced Cancer Diagnostics, Nagoya University Graduate School of Medicine, Nagoya 466-8550, Japan

**Keywords:** rectal cancer, neoadjuvant chemoradiation, mouse model, proteomics, biomarkers

## Abstract

**Simple Summary:**

Although pathologic complete response (pCR) to neoadjuvant chemoradiation (nCRT) in locally advanced rectal cancer (LARC) is associated with better outcomes, a subset of tumors exhibit resistance to nCRT. Therefore, there is a need of biomarkers to predict the nCRT response and increment efforts for personalized therapeutic options. To this end, we analyzed pretreatment plasma proteome of a mouse model of rectal cancer treated with concurrent chemoradiation, resulting in identification and validation of plasma VEGFR3 as a potential predicting biomarker. In addition, plasma levels of EGFR and COX2, previously validated tissue-based predicting biomarkers, were significantly higher in non-pCR than pCR LARC patients, indicating that EGFR and COX2 can also serve as blood-based biomarkers. The performance of the biomarker panel combining VEGFR3, EGFR, and COX2 were significantly improved compared to that of each marker alone, providing a rationale for further integration and refinement of the biomarker panel and validation in the larger sample sets.

**Abstract:**

The current standard of care for patients with locally advanced rectal cancer (LARC) is neoadjuvant chemoradiation (nCRT) followed by total mesorectal excision surgery. However, the response to nCRT varies among patients and only about 20% of LARC patients achieve a pathologic complete response (pCR) at the time of surgery. Therefore, there is an unmet need for biomarkers that could predict the response to nCRT at an early time point, allowing for the selection of LARC patients who would or would not benefit from nCRT. To identify blood-based biomarkers for prediction of nCRT response, we performed in-depth quantitative proteomic analysis of pretreatment plasma from mice bearing rectal tumors treated with concurrent chemoradiation, resulting in the quantification of 567 proteins. Among the plasma proteins that increased in mice with residual rectal tumor after chemoradiation compared to mice that achieved regression, we selected three proteins (Vascular endothelial growth factor receptor 3 [VEGFR3], Insulin like growth factor binding protein 4 [IGFBP4], and Cathepsin B [CTSB]) for validation in human plasma samples. In addition, we explored whether four tissue protein biomarkers previously shown to predict response to nCRT (Epidermal growth factor receptor [EGFR], Ki-67, E-cadherin, and Prostaglandin G/H synthase 2 [COX2]) also act as potential blood biomarkers. Using immunoassays for these seven biomarker candidates as well as Carcinoembryonic antigen [CEA] levels on plasma collected before nCRT from 34 patients with LARC (6 pCR and 28 non-pCR), we observed that levels of VEGFR3 (*p* = 0.0451, AUC = 0.720), EGFR (*p* = 0.0128, AUC = 0.679), and COX2 (*p* = 0.0397, AUC = 0.679) were significantly increased in the plasma of non-pCR LARC patients compared to those of pCR LARC patients. The performance of the logistic regression model combining VEGFR3, EGFR, and COX2 was significantly improved compared with the performance of each biomarker, yielding an AUC of 0.869 (sensitivity 43% at 95% specificity). Levels of VEGFR3 and EGFR were significantly decreased 5 to 7 months after tumor resection in plasma from 18 surgically resected rectal cancer patients, suggesting that VEGFR3 and EGFR may emanate from tumors. These findings suggest that circulating VEGFR3 can contribute to the prediction of the nCRT response in LARC patients together with circulating EGFR and COX2.

## 1. Introduction

The current standard of care for patients with clinical stage II or III locally advanced rectal cancer (LARC), defined as T3–T4 or node-positive non-metastatic disease, is neoadjuvant chemoradiation (nCRT) followed by total mesorectal excision (TME) surgery to improve resectability, anal sphincter preservation, and long-term outcome [1,2]. However, the response to nCRT in LARC varies among patients. After nCRT, about 20% of LARC patients achieve a pathological complete response (pCR), which is associated with favorable 5-year disease-free survival compared to those without complete response (non-pCR) [3,4]. Conversely, while ~40% of LARC patients achieve a wide range of partial responses, a subset (~20%) of tumors exhibit resistance to nCRT, demonstrating either progression or only minimal regression/stable disease [5].

Given the achievement of pCR in a significant proportion of patients undergoing nCRT and the adverse effects of major TME surgery such as perioperative mortality, anastomotic leak, stoma-related complications, and long-term urinary and sexual dysfunction [6,7,8], there is a growing interest in organ preservation for LARC patients who achieve a clinical complete response (cCR) after nCRT. Since 2004, a series of studies have reported the promising potential of a watch-and-wait strategy to avoid major TME surgery [9,10,11,12]. Recent large datasets from meta-analyses and registry studies indicated that 5-year overall survival did not differ between patients treated with a watch-and-wait and those with surgery [13,14], suggesting that the watch-and-wait strategy can be an alternative to TME surgery with low oncological risk. However, the significant limitation of the watch-and-wait strategy is the poor concordance between pCR and cCR, which can result in local regrowth after nCRT [13,14,15]. Therefore, there is an immense need for biomarkers for safe adoption of the watch-and-wait strategy to predict pCR and identify patients who may potentially avoid surgery after completion of nCRT.

A wide variety of clinical, pathologic, and radiologic factors, including tumor size, differentiation, clinical T and N stages, and tumor regression rates, have been associated with nCRT response [16,17,18,19]. Several potential tissue- or blood-based molecular biomarkers to predict the nCRT response, including DNA methylation, protein, miRNA, and cfDNA, have also been described [20,21,22,23]. As carcinoembryonic antigen (CEA) is routinely used for disease monitoring in colorectal cancer, the relationship of CEA and the nCRT response has been most widely studied. A recent meta-analysis suggest that pretreatment CEA levels were significantly and inversely correlated with the rate of pCR [24]. However, to date, none of these clinicopathological, radiological, or molecular biomarkers have yet reached the clinic due to inadequate sensitivity and specificity.

In this study, we sought to identify blood-based biomarkers that can differentiate patients who will achieve a pCR versus non-pCR by analyzing the plasma proteome of a mouse model with rectal cancer that recapitulate molecular and biological features of human rectal cancer [25]. This approach allows minimization of extraneous variability and blood sampling at defined pre-therapeutic time points during the course of tumor progression [26]. In addition, we investigated whether previously validated tissue-based biomarkers for nCRT response [20] can also serve as blood-based biomarkers for predicting pCR.

## 2. Materials and Methods

### 2.1. Mouse Model

All animal experiments were conducted in accordance with institutional and national guidelines and regulations with approval by the Institutional Animal Care and Use Committee at The University of Texas MD Anderson Cancer Center. At 8 weeks of age, mice were administered with a 4-hydroxytamoxifen (4-OHT) enema (1 mg/mL) and doxycycline [25]. Beginning at 4 weeks post-induction, tumor size was measured as the percentage of tumor occlusion of the lumen by weekly colonoscopy. Once the percentage of tumor occlusion of the lumen reached 50%, intraperitoneal injection of 5-FU (30 mg/kg) and concurrent radiation (5 Gy per fraction) were administered to four mice with rectal tumors for 5 consecutive days, resulting in two mice showing complete tumor regression and two mice with residual tumors. Four control mice at the same age without Cre DNA recombinase were also treated with the same regimen. Five days prior to the treatment, plasma was collected, and Magnetic resonance imaging (MRI) was performed to assess rectal tumors. Mice were euthanized a week after chemoradiation and response to chemoradiation was pathologically evaluated.

### 2.2. Mass Spectrometry Analysis of Pretreatment Mouse Plasma Samples

An independent pool of pretreatment plasmas from two mice with rectal tumors achieving regression, two mice with residual rectal tumors after chemoradiation, and four control mice were created. Each pool of mouse plasma was subjected to immunodepletion, whereby the top three abundant proteins (albumin, IgG, and transferrin) were removed using an Immunodepletion column (Agilent Technologies, Santa Clara, CA, USA). The remaining low abundant proteins in each sample were treated with 25 mM tris(2-carboxyethyl)phosphine (TCEP) for Cys reduction and subsequently labeled with Iodoacetyl Tandem Mass Tag (IodoTMT) sixplex isobaric label reagent (Thermo Fisher Scientific, Waltham, USA). The mixture of labeled samples was separated by an orthogonal two-dimensional high-performance liquid chromatography (2D-HPLC) system (Shimadzu, Kyoto, Japan) with eight fractions of anion-exchange (Agilent Technologies, Santa Clara, USA) as the first dimension followed by 12 reversed-phase fractions by RPGS reversed-phase column (4.6 mm I.D. × 150 mm, 15 µm, 1000 Å, Column Technology Inc, Fremont, CA, USA) as the second dimension. Collected protein fractions were lyophilized, digested with trypsin, and analyzed by nano LC–high definition MS^E^ (HDMS^E^) with Synapt G2Si ion-mobility quadrupole time-of-flight (Q-TOF) mass spectrometry (Waters, Milford, USA).

The 2 h gradient elution was performed in a capillary column (C18, 3 µm 120 Å, 75µmID × 25 cmL, Column Technology, Inc, Fremont, USA) at 500 nl/min with the mobile phase A 0.1% formic acid (FA) in 2% acetonitrile (ACN) and B 0.1% FA in 98% ACN. The mass spectrometer was operated with a resolving power of at least 20,000 full width at half maximum (FWHM) at m/z 785.843 (+2, Glu1-fibrinopeptide B) nano electrospray ionization (ESI) source with a NanoLockSpray. The lock mass channel was sampled every 60 s.

Accurate LC-HDMS^E^ data were acquired in an alternating low energy (HDMS) and high energy (HDMS^E)^ mode with mass scan range from m/z 50 to 1800 under a capillary voltage of 2.8 kV, a source temperature of 100 °C, and a cone voltage of 30 V. The spectral acquisition in each mode is 1.0 s with a 0.1 s inter-scan. In HDMS mode, data are collected at a collision energy of 2 eV in both Trap and Transfer cell. In HDMS^E^ mode, the collision energy is ramped up from 25 to 55 eV in the Transfer cell. The acquired data were processed through ProteinLynx Global Server (PLGS, WATERS, Milford, USA) and searched against the Uniprot mouse database at 4% false discovery rate (FDR). The identified proteins were filtered with ≤ 5 ppm mass accuracy of sequenced peptides. Quantile normalization approach was used to normalize the peak intensities of reporter ions before protein quantification.

### 2.3. Human Plasma Samples

All human plasma samples were obtained following Institutional Review Board approval and informed consent. Plasma samples were collected from 34 treatment-naïve LARC patients undergoing neoadjuvant chemoradiation (50.4 Gy in 28 daily fractions of 1.8 Gy with concurrent capecitabine (an oral prodrug of 5-fluorouracil)) at the MD Anderson Cancer Center, and used for validation of biomarker candidates (pretreatment LARC set). After completion of chemoradiation, patients underwent surgical excision, and standardized pathological procedures were followed for the assessment of residual disease. An independent set of plasma samples collected at the time of diagnosis and 5 to 7 months after surgery from 18 surgically resected rectal cancer patients at the Aichi Cancer Center was used to assess the association between biomarker candidates and surgical resection (pre-and post-surgery RC set). All patients in the pre- and post-surgery RC set were treated by surgery alone.

### 2.4. Luminex Assays

Levels of Vascular endothelial growth factor receptor 3 (VEGFR3), Insulin like growth factor binding protein 4 (IGFBP4), Carcinoembryonic antigen (CEA), Epidermal growth factor receptor (EGFR), and E-cadherin were measured using the Luminex kit (HANG2MAG-12K, HIGFBMAG-53K, HCCBP1MAG-58K, and HSCRMAG-32K from Millipore, and EPX010-12315-901 from Life Technologies), according to the manufacturer’s instructions. Each sample was assayed in duplicate, and the absorbance was measured with a calibrated Bio-Plex machine (Bio-Plex MAGPIX System, Bio-Rad, Hercules, CA, USA).

### 2.5. ELISA Assays

Levels of Cathepsin B (CTSB), Ki-67, and Prostaglandin G/H synthase 2 (COX2) were determined using enzyme-linked immunosorbent assay (ELISA) kits (ab119584 from Abcam, Cambridge, UK; DY7617-05 from R&D Systems, Minneapolis, MN, USA; and RAB1034-1KT from Sigma-Aldrich, St. Louis, MO, USA) and according to the manufacturer’s protocol. For all ELISA experiments, each sample was assayed in duplicate, and the absorbance was measured with a FLUOstar Omega microplate reader (BMG Labtech, Ortenberg, Germany).

### 2.6. Statistical Analysis

Categorical data were compared by Fisher’s exact test or a chi-square test using Prism 7.05/e software (GraphPad). For all Luminex and ELISA assays, an internal control sample was run in every plate, and each value of the samples was divided by the mean value of the internal control in the same plate to correct the interplate variability. Individual biomarker performance was assessed using the Welch’s t-test or the paired t-test. Sensitivity, specificity, and the area under the curve (AUC) were determined by a receiver operating characteristic analysis. The likelihood ratio test was employed to assess the significance of the model based on the biomarker panel combining VEGFR3, EGFR, and COX2.

## 3. Results

### 3.1. Proteomic Profiling of Pretreatment Plasmas from a Mouse Model of Rectal Cancer Treated with Chemoradiation

**Figure 1:**
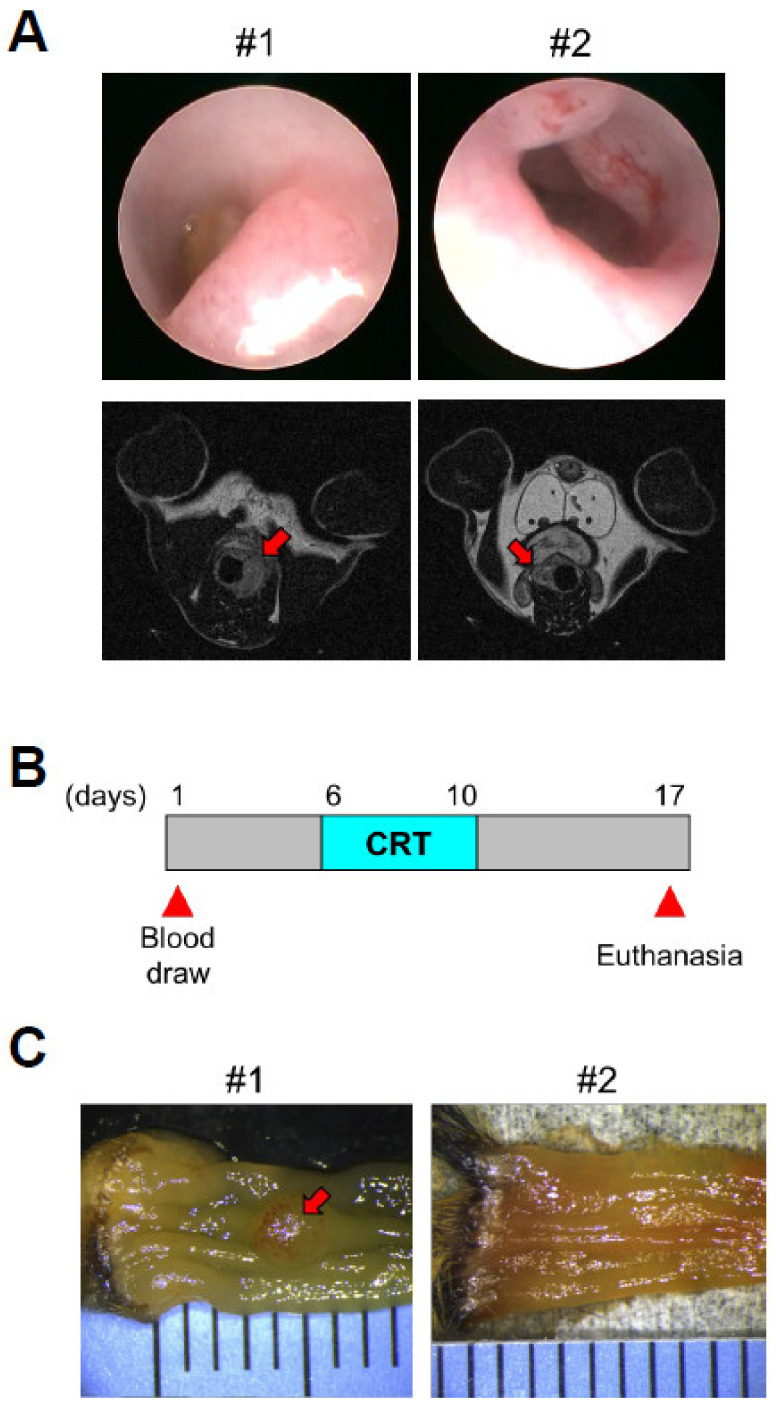
Chemoradiation treatment of iKAP mice with rectal tumors. (**A**). Endoscopic images (top) and MRI scans (bottom) of rectal tumors. Red arrows in MRI scans indicate rectal tumors. (**B**). Outline of the experimental design for intraperitoneal injection of 5-FU and concurrent radiation. (**C**). Macroscopic images of colons of iKAP mice with residual tumor (Non-regression) (#1) and with complete tumor regression (Regression) (#2). A red arrow indicates residual tumors. CRT: chemoradiation.

To faithfully recapitulate the development of human locally advanced rectal cancer (LARC), we utilized a genetically engineered mouse model of colorectal cancer, which harbors a Doxycycline (Dox)-inducible oncogenic Kras allele and conditional null alleles of Apc and Trp53 (iKAP) [25]. Approximately 25% of iKAP mice are expected to develop only rectal tumors by colon-specific activation of Cre DNA recombinase with rectal enema of 4-OHT. Occurrence of rectal tumors in Dox-treated iKAP mice was confirmed by endoscopy, MRI, and biopsy (Figure 1A). iKAP mice with rectal tumors and control mice without Cre DNA recombinase received intraperitoneal injection of 5-FU (30 mg/kg) and concurrent radiation (5 Gy per fraction) for 5 consecutive days (Figure 1B). To assess the response to chemoradiation, mice were sacrificed a week after the treatment. Figure 1C depicts macroscopic images of colons of iKAP mice with residual tumors (non-regression) (#1) and with complete tumor regression (Regression) (#2), while rectal tumors of #1 and #2 mice were not distinctively different before chemoradiation (Figure 1A).

**Figure 2:**
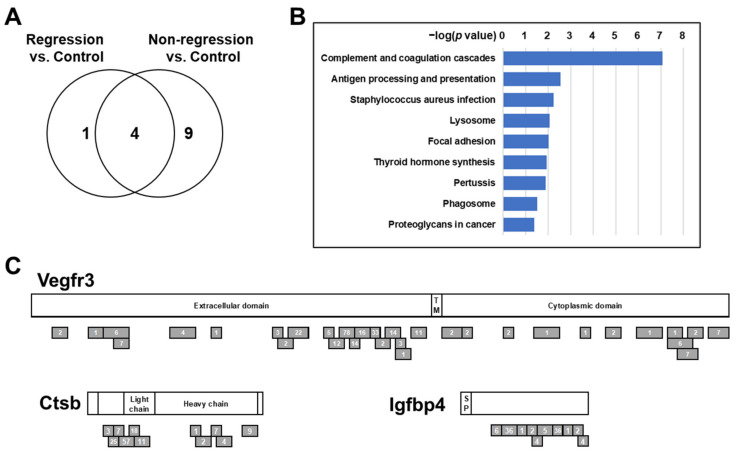
Proteomic analysis of pretreatment plasmas from iKAP mice with complete tumor regression and with residual tumors. (**A**). Venn diagrams of pathways are significantly associated with more than a two-fold increase of proteins in the plasma of Regression mice or Non-regression mice compared to control mice. (**B**). Pathways uniquely identified in the comparison of Non-regression vs. control. *p* values were calculated by hypergeometric test. (**C**). Schema of mouse Vegfr3, Ctsb, and Igfbp4. Gray bars indicate peptides identified in mouse plasma. Numbers indicate mass spectra counts for each peptide. The amino acid sequences are based on P35917-1 for Vegfr3, P10605-1 for Ctsb, and P47879-1 for Igfbp4.

To identify plasma proteins that can predict pCR before chemoradiation, plasma samples were collected 5 days prior to chemoradiation from iKAP mice with rectal tumors and control mice (Figure 1B). For mass spectrometry analysis, plasma samples from two Regression mice, two Non-regression mice, and four control mice were respectively pooled. In-depth quantitative proteomic analysis with using tandem mass tags (TMT) labeling resulted in quantification of 567 proteins (393 unique genes). To gain insights into the underlying biological difference of increased plasma proteins in Regression mice and Non-regression mice, we performed a pathway analysis of proteins with more than a two-fold increase in the plasma of either Regression mice or Non-regression mice compared to control mice using WebGestalt (http://www.webgestalt.org/, accessed on 25 March 2020) [27]. Over-Representation Analysis based on KEGG (https://www.genome.jp/kegg/, accessed on 25 March 2020) [28] resulted in identification of 5 and 13 pathways that were significantly associated with increased proteins in the plasma of Regression mice or Non-regression mice compared to control mice, respectively (*p* values < 0.05, hypergeometric test, and with three or more overlapping proteins) (Figure 2A and Supplementary Table S1). While four pathways commonly identified in the comparison of Regression vs. control and Non-regression vs. control were associated with metabolism, nine pathways that were uniquely identified in the comparison of Non-regression vs. control were associated with the immune system and infectious disease (Complement and coagulation cascades; Antigen processing and presentation; Staphylococcus aureus infection; Pertussis), intracellular transport and catabolism (Lysosome; Phagosome), focal adhesion (Focal adhesion; Proteoglycans in cancer), and the endocrine system (Thyroid hormone synthesis) (Figure 2A,B).

To determine biomarker candidates for prediction of pCR, we applied the following criteria: (1) the number of quantified peptides ≥ 10, (2) Non-regression/Control ratio > 3, and (3) Non-regression/Regression ratio > 3. Nine proteins passed these criteria (Table 1), and intriguingly, Collagen type I alpha 1 chain (Col1a1), Cathepsin B (Ctsb), and Vascular endothelial growth factor receptor 3 (Vegfr3) were included in the pathways uniquely associated with increased plasma proteins in Non-regression mice (Supplementary Table S1), suggesting a possible biological link between these proteins and rectal tumors resistant to chemoradiation. In addition, Insulin like growth factor binding protein 4 (Igfbp4) is of interest, as it can bind to and modulate the function of Insulin-like growth factor I (Igf1) [29], which is also included in two pathways associated with focal adhesion (Supplementary Table S1). Our mass spectrometry analysis yielded substantial peptide coverage for Vegfr3, Ctsb, and Igfbp4 (Figure 2C and Table 1), and therefore, we selected these three proteins for validation in human plasma samples.

### 3.2. Validation of Protein Biomarker Candidates in a Set of LARC Plasma Samples

VEGFR3, CTSB, and IGFBP4, as well as Carcinoembryonic antigen (CEA) were assayed in the pretreatment LARC set consisting of plasma samples collected prior to nCRT from 34 patients with LARC. In the pretreatment LARC set, 6 (17.6%) patients achieved pCR (pCR) and 28 (82.4%) patients had residual tumors after nCRT (non-pCR). While non-pCR LARC patients had more advanced T stages, clinical factors did not show a statistically significant difference between pCR and non-pCR LARC patients (Table 2).

In addition to three selected biomarker candidates, as many potential tissue-based biomarkers have been associated with nCRT response [20], we sought to determine whether tissue-based protein biomarkers in blood also can have the potential to predict pCR. According to prior studies, we selected four proteins, including Epidermal growth factor receptor (EGFR), Ki-67, Prostaglandin G/H synthase 2 (COX2), and E-cadherin, for testing in the pretreatment LARC set.

**Figure 3:**
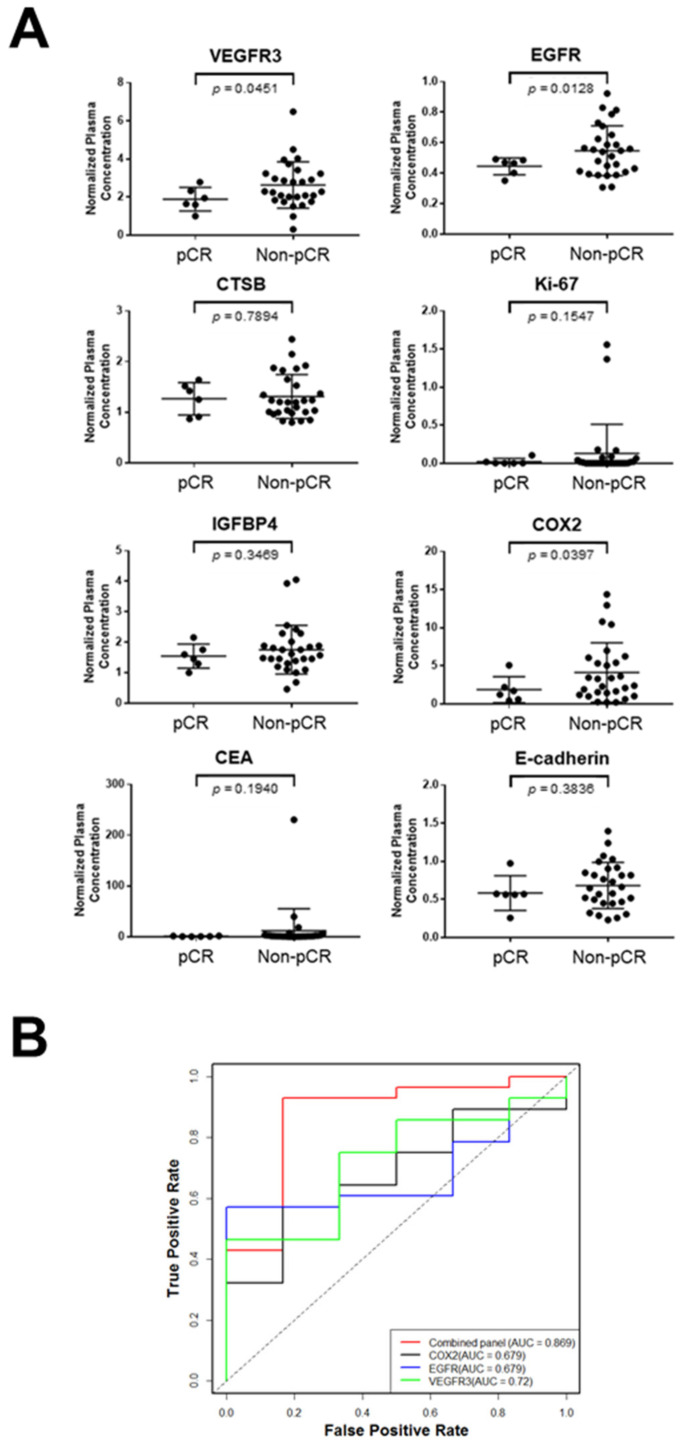
Validation of biomarker candidates in the pretreatment LARC set. (**A**). Levels of VEGFR3, CTSB, IGFBP4, CEA, EGFR, Ki-67, COX2, and E-cadherin in plasmas collected before nCRT from LARC patients who achieved pCR (pCR; *n* = 6) and LARC patients who had residual tumors after nCRT (non-pCR; *n* = 28). Horizontal lines indicate mean and standard deviation. *p* values were calculated using Welch’s t-test. (**B**). Receiver operating characteristic curves for VEGFR3, EGFR, COX2, and their combination in the pretreatment LARC set. CEA: Carcinoembryonic antigen, EGFR: Epidermal growth factor receptor, COX2: Prostaglandin G/H synthase 2, AUC: the area under the curve.

Plasma levels of VEGFR3, EGFR, and COX2 were significantly higher in non-pCR LARC compared to pCR LARC (VEGFR3: *p* = 0.0451, EGFR: *p* = 0.0128, COX2: *p* = 0.0397, Welch’s t-test) (Figure 3A). CEA and other four biomarker candidates were not significantly different between pCR and non-pCR LARC patients (CEA: *p* = 0.1940, CTSB: *p* = 0.7894, IGFBP4: *p* = 0.3469, Ki-67: *p* = 0.1547, E-cadherin: *p* = 0.3836, Welch’s *t*-test).

We explored whether a combination rather than individual biomarkers allows for better discrimination of pCR and non-CR. The combination of VEGFR3, EGFR, and COX2 using logistic regression yielded an area under the curve (AUC) of 0.869 with a sensitivity of 43% at 95% specificity in the comparison of pCR and non-pCR LARC patients, which was significantly higher than AUC of each marker alone (VEGFR3: AUC = 0.720, *p* = 0.0152, likelihood ratio test; EGFR: AUC = 0.679, *p* = 0.0160, likelihood ratio test; COX2: AUC = 0.679, *p* = 0.0151, likelihood ratio test) in the pretreatment LARC set (Figure 3B).

### 3.3. Correlation of VEGFR3, EGFR, and COX2 in the Plasma of Rectal Cancer Patients Before and After Surgery

**Figure 4:**
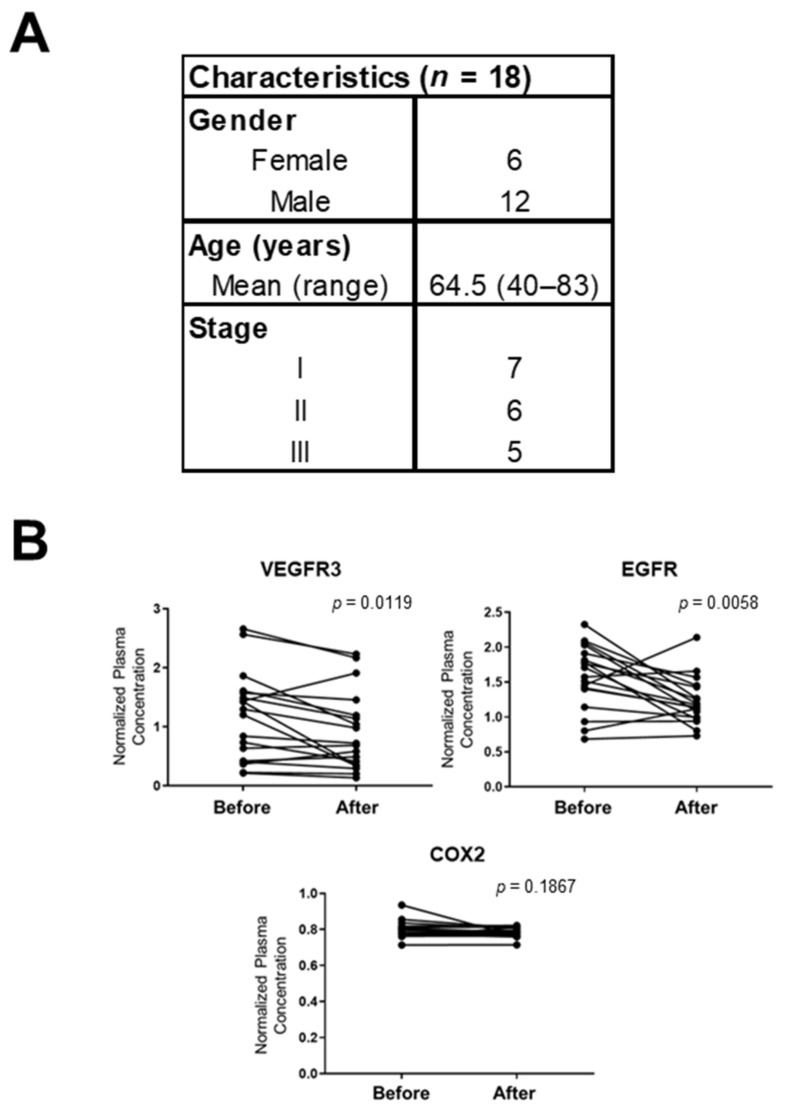
Correlation of plasma VEGFR3, EGFR, and COX2 with surgical resection in patients with rectal cancer. (**A**). Subject characteristics in the pre-and post-surgery RC set. (**B**). Levels of VEGFR3, EGFR, and COX2 in plasmas collected at the time of diagnosis (Before) and 5 to 7 months after surgery (After) from 18 rectal cancer patients. *p* values were calculated by paired t-tests.

We next determined whether plasma levels of VEGFR3, EGFR, and COX2 are associated with surgical resection of rectal cancer. For this analysis, VEGFR3, EGFR, and COX2 were assayed in an independent set of plasma consisting of samples collected from 18 rectal cancer patients at the time of diagnosis and 5 to 7 months after surgical resection (pre-and post-surgery RC set; Figure 4A). All these rectal cancer patients remained disease-free for five years after surgery. Plasma levels of VEGFR3 and EGFR were significantly decreased after tumor resection (VEGFR3: *p* = 0.0119, EGFR: *p* = 0.0058, paired t-test) (Figure 4B). Levels of plasma COX2 did not change significantly after surgery compared to presurgical levels (*p* = 0.1867, paired t-test).

## 4. Discussion

In this study, we profiled the pretreatment plasma proteome of a mouse model of rectal cancer treated with concurrent chemoradiation to identify potential blood-based biomarkers for predicting pCR in LARC patients. Among plasma protein signatures associated with Non-regression, proteins involved in focal adhesion are of particular interest, as deregulation of integrin-mediated focal adhesion has been shown to lead to therapeutic resistance [30]. We found that levels of Vegfr3, one of the proteins in the “Focal adhesion” pathway, was significantly increased in the pretreatment plasma of Non-regression mice compared to Regression mice and control mice. Increased levels of circulating VEGFR3 were validated in plasma collected prior to nCRT from non-pCR LARC patients compared to pCR LARC patients. We also demonstrated that plasma VEGFR3 levels were significantly decreased after surgical resection of rectal tumors. Yeh et al. recently showed a significant correlation between circulating VEGFR3 levels and expression of VEGFR3 in tumor tissues [31]. These findings suggest that circulating VEGFR3 emanated from tumor tissues. VEGFR3 is crucial for the development and maintenance of blood and lymphatic vascular systems [32]. While VEGFR3 is primarily expressed in lymphatic endothelial cells, VEGFR3 and its main ligand VEGF-C are expressed in tumor cells of various types of cancer, including colorectal cancer [33]. Higher expression of VEGFR3 in tumor tissues has been also associated with advanced TNM stages, the occurrence of metastasis, and poor prognosis in colorectal cancer [31,34]. Recent studies have revealed that activation of VEGF-C/VEGFR3 signaling promotes tumor growth and invasion by disrupting the lymphatic endothelial barrier and by recruiting and inducing immunosuppressive tumor-associated macrophages in colorectal cancer [34,35]. Given the emerging evidence suggesting crucial roles of VEGF-C/VEGFR3 axis in cancer progression, multiple therapeutic strategies for VEGF-C/VEGFR3-targeted therapies, including small molecule VEGFR3 inhibitors, monoclonal antibodies targeting VEGF-C, and neutralizing antibodies or peptides that block VEGFR3 signaling, have been developed [33]. Therefore, while our findings indicated the potential of circulating VEGFR3 as a biomarker for nCRT response, circulating VEGFR3 may also serve as a biomarker for the prediction of prognosis and response to VEGF-C/VEGFR3-targeted therapies.

In this study, we also explored whether tissue-based biomarkers that have been associated with nCRT response can serve as blood-based biomarkers for pCR prediction. Among four tissue-based biomarkers selected for testing, we observed significantly increased levels of circulating EGFR and COX2 in plasmas collected prior to nCRT from non-pCR LARC patients compared to pCR LARC patients.

Several studies have reported the significant association of tissue EGFR expression and response to nCRT in LARC [36]. A recent study indicated that higher expression of EGFR in the nucleus is associated with poor survival in LARC patients treated with nCRT [37]. While we demonstrated for the first time that circulating EGFR could be used as a potential biomarker for predicting pCR in rectal cancer, circulating EGFR has been associated with response to therapy in several types of cancer [38]. Interestingly, Okada et al. demonstrated that downregulation of EGFR in tumor tissue after treatment with anti-EGFR antibodies was significantly correlated with therapeutic response in patients with colorectal cancer [39]. Although a recent meta-analysis indicated the addition of EGFR inhibitors did not improve the efficacy of neoadjuvant therapy in KRAS-wild type LARC patients [40], monitoring circulating EGFR may help determine whether to continue EGFR-targeted therapy or not.

Regarding tissue COX2 expression in LARC, previous studies have reported an association of COX2 overexpression and poor response to nCRT [20]. While we demonstrated that circulating COX2 was significantly increased in the plasma of non-pCR LARC patients compared to pCR LARC patients, surgical resection of rectal tumors did not affect circulating COX2 levels. As COX2 is induced by various inflammation mediators [41], increased levels of circulating COX2 in LARC patients may be due to cancer-associated systemic inflammation or some other physiological stress, such as infection. However, given that development of cancer-associated systemic inflammation is associated with a poorer outcome [42] and that a recent phase II clinical trial of nCRT combining COX2 inhibitor celecoxib in LARC improved efficacy and decreased toxicity [43], it would be interesting to determine whether circulating COX2 levels can predict prognosis and response to nCRT combined with COX2 inhibitors.

The logistic regression model combining circulating VEGFR3, EGFR, and COX2 yielded a significantly higher AUC in differentiating pCR and non-CR compared to that of each marker alone. However, blood contains a wide variety of measurable molecules and cellular materials, including exosomes, tumor-derived DNAs, microRNAs, autoantibodies, and metabolites, making blood a rich resource of biomarkers. Therefore, it is critical to determine the relevance and relative contributions of the different types of biomarkers in the same samples [20], allowing to further refine the biomarker panel with integrating other previously or newly identified biomarkers. In addition, due to a small sample size in the current study, the performance of these three blood-based biomarkers will need to be assessed in larger sample sets, and the biomarker panel will need to be further refined with integrating other previously validated biomarkers.

## 5. Conclusions

In conclusion, we identified circulating VEGFR3 as a novel biomarker for predicting pCR through proteomic analysis of plasmas from a mouse model of rectal cancer, and further confirmed increased VEGFR3 levels in pretreatment plasmas from non-pCR LARC patients compared to pCR LARC patients. We also demonstrated that levels of circulating EGFR and COX2, known tissue-based biomarkers for nCRT response, were significantly increased in pretreatment plasma of non-pCR LARC patients. Our findings provide a rationale for further studies to safely adopt the watch-and-wait strategy with using blood-based biomarkers in LARC patients.

## Figures and Tables

**Table 1:** Plasma proteins increased in Non-regression iKAP mice.

Protein	Total Number of Peptides	Non-Regression/Control Ratio	Non-Regression/Regression Ratio
Igfbp4	38	9.9	3.6
Col1a1	13	8.1	3.7
Prdx6	10	6.4	5.2
Park7	13	4.9	5.0
F13b	138	4.2	3.7
Flna	20	4.1	3.4
Ctsb	44	4.0	3.0
Vegfr3	91	3.8	3.7
Blvrb	14	3.3	4.4

Igfbp4: Insulin like growth factor binding protein 4, Col1a1: Collagen type I alpha 1 chain, Prdx6: Peroxiredoxin-6, Park7: Parkinson disease protein 7, F13b: Coagulation factor XIII B chain, Flna: Filamin-A, Ctsb: Cathepsin B, Vegfr3: Vascular endothelial growth factor receptor 3, Blvrb: Biliverdin reductase B.

**Table 2:** Subject characteristics in the pretreatment LARC set.

Characteristics	pCR (*n* = 6)	Non-pCR (*n* = 28)	*p* Value
**Gender**			
Female	3	13	>0.9999
Male	3	15	
**Age (years)**			
Mean (range)	56.0 (45–67)	56.5 (28–74)	
<56	3	12	>0.9999
≥56	3	16	
**T stage**			
T2	3	3	0.0603
T3	3	21	
T4	0	4	
**N stage**			
N0	0	1	0.2528
N1	6	18	
N2	0	8	
**Stage**			
IIa	0	1	0.1379
IIIa	3	3	
IIIb	3	22	
IIIc	0	2	
**Tumor length (cm)**			
<4.5	2	13	0.6722
≥4.5	4	15	
**Distance from anal verge (cm)**			
<6.5	4	13	0.6562
≥6.5	2	15	

*p* values were calculated by Fisher’s exact test or a chi-square test.

## Data Availability

The results of the proteomic analysis of mouse plasmas presented in this study are available upon reasonable request from the corresponding author.

## Supplementary Materials

The following are available online at https://www.mdpi.com/article/10.3390/cancers13143642/s1. Table S1: Significantly associated pathways for more than a two-fold increase of proteins in the plasma of pCR mice or non-pCR mice compared to control mice.
